# A cost-utility analysis of newborn screening for spinal muscular atrophy in Canada

**DOI:** 10.1186/s13023-025-03927-6

**Published:** 2025-08-13

**Authors:** Alex Pace, Weston Roda, Corrina Poon, Hugh J. McMillan, Maryam Oskoui, Alex MacKenzie, Pranesh Chakraborty, Jeff Round

**Affiliations:** 1 Children’s Hospital of Eastern Ontario, Ottawa, ON Canada; 2 Institute of Health Economics, Edmonton, AB Canada; 3 McGill University Health Centre, Montreal, QC Canada; 4 University of Alberta, Edmonton, AB Canada

**Keywords:** Spinal muscular atrophy, Newborn screening, Economic evaluation, Cost-Utility analysis, Markov model, Decision tree, Canadian health system

## Abstract

**Background:**

Spinal muscular atrophy (SMA) is a neuromuscular disorder caused by the loss of the *SMN1* gene, with an estimated birth prevalence of about 1 in 10,000. Early intervention with disease-modifying therapies (DMTs) significantly improves outcomes. This study evaluates the economic implications and health benefits of newborn screening (NBS) for SMA in Canada from the societal perspective.

**Methods:**

A decision analytic model was developed, which combined a decision tree for the screening algorithm and a Markov model for long-term health outcomes. The Markov model included health states based on WHO motor milestones. The population cohort of 357,903 live newborns reflects the 2022–2023 births in Canada. Screening is performed on dried blood spot testing which evaluates for biallelic deletions in *SMN1*. Cost inputs encompassed treatment and health state costs, while utility values reflected quality of life in each health state.

**Results:**

NBS for SMA is expected to identify 37.1 (95% CI: 15.0, 70.7) newborns annually in Canada. Our analysis over a lifetime horizon and a discount rate of 1.5% shows NBS and early treatment has an incremental cost of -$146,187,000 (95% CI: -249,773,777 to − 17,890,034) and incremental benefit of 872 (95% CI: -193, 2329) quality-adjusted life years (QALYs) compared to no NBS and late treatment. This resulted in a mean ICER value of -$173,572/QALY.

**Conclusion:**

The decision analytic model indicated that overall NBS is cost-saving and more effective than no NBS and late treatment in the Canadian health system.

**Supplementary Information:**

The online version contains supplementary material available at 10.1186/s13023-025-03927-6.

## Background

Spinal muscular atrophy (SMA) is a severe autosomal recessive neuromuscular disorder resulting from the loss of the survival motor neuron 1 (*SMN1*) gene. This genetic defect leads to the progressive degeneration of motor neurons, causing significant muscle weakness and atrophy. Homozygous deletion of *SMN1* is estimated to cause 95% of SMA cases, while the remaining 5% result from pathogenic *SMN1* missense or nonsense mutations [1].

SMA is classified into several types based on the severity and onset of symptoms. The most severe form, SMA type 1, manifests within the first six months of life, leading to rapid progression of muscle weakness, respiratory failure, and often death if untreated in the first two years of life. SMA type 2 is characterized by symptom onset occurring within the first 18 months of life. Patients with SMA type 2 can typically sit unassisted, although in the absence of treatment are not expected to walk unassisted. Symptoms of SMA type 3 generally begin after 18 months of life, with children eventually achieving the ability to walk at some point [1, 2].

The incidence of SMA is approximately 1 in 10,000 live births, with a prevalence of 1 to 2 per 100,000 individuals [3, 4]. Although recent Canadian pilot studies have reported varying incidence rates of SMA, ranging from 1 in 9,000 to 1 in 27,000, the short duration of these screening programs makes it challenging to determine the true incidence of SMA in Canada [5, 6]. Recent advancements in disease-modifying therapies (DMTs) have significantly altered the prognosis for individuals with SMA. Three DMTs: nusinersen (Spinraza), onasemnogene abeparvovec (Zolgensma) (OA), and risdiplam (Evrysdi) have been approved for use, and reimbursement in Canada. Clinical trials have shown that earlier intervention with these therapies leads to better outcomes, including improved motor function and achievement of developmental milestones [7–9]. These clinical trials also demonstrated that presymptomatic treatment has the greatest impact on survival and motor milestone development, with the greatest benefit coming when treatment is initiated before the first symptom [10].

In Canada, SMA screening has been increasingly adopted, with ten of the thirteen provinces and territories implementing the program, covering 95.56% of Canadian newborns to date [11]. Despite the evident clinical benefits, the economic implications of widespread newborn screening (NBS) for SMA are currently unknown in Canada. To address this gap, this study explored the lifetime costs and health effects of NBS for SMA with early treatment versus no NBS and late treatment within the Canadian context.

## Methods

### Study aim

This study aimed to estimate the lifetime costs and health effects of NBS for SMA with early treatment versus no NBS and late treatment, within the Canadian context from the societal perspective.

### Model structure

We developed a decision analytic model to estimate the cost-utility of NBS for SMA. The model structure is a hybrid decision tree and Markov model. The decision tree component represents the screening algorithm for newborns in Canada, capturing the costs associated with screening. The Markov component is a state-transition model to estimate the longer-term health outcomes and costs of living with SMA.

The Markov model health states were developed using the World Health Organization’s (WHO) motor milestone achievements of healthy infants [12]. The model includes the following health states: permanent assisted ventilation (PAV) (State E), not-sitting (State D), sitting (State C), walking (State B), broad range of normal development (BRND) (State A), and death (State F). These health states are consistent with previous SMA Markov Models [13, 14]. Patients identified through NBS enter the model in the not-sitting state at 1 month of age as they have not achieved any motor milestones to date; this is when they would be first eligible to receive treatment. The costs applied in the not-sitting state depend on the age of the patient. A patient is expected to sit by 9 months of age, according to WHO motor milestone 99th percentile of achievement [12]. If the patient is less than or equal to 9 months of age, then the BRND (State A) costs and utility are applied, as this is considered normal development. If the patient is greater than 9 months of age, then the non-sitting costs, which include medical visits and caregiver costs, and utility are applied. Similarly, since a patient is expected to walk by 18 months of age, in the sitting and walking states, BRND costs and utility are applied if the patient is less than or equal to 18 months. Those who were not screened, or missed by screening, enter the model in different states depending on the severity of their disease.

Patients with SMA type 1 who clinically present enter the model in the not-sitting state at 3.9 months of age. Patients with SMA type 2 who clinically present enter the sitting state at 4.4 years of age. Patients with SMA type 3 who clinically present enter the walking (76%) or sitting state (24%) at 8.9 years of age. The ages of clinical presentation were determined from clinical trials [15, 16]. Clinical trial evidence was also used to estimate the transition probabilities through the Markov model. Details of the estimation of transition probabilities are described further in the Clinical Inputs section. A 1-month cycle length was used in the Markov model to most accurately capture motor milestone changes of children over time. A lifetime horizon was modeled for the base case analysis, with the model running for 80 years (960 months). Costs and health outcomes were both discounted at 1.5% [17].

### Population cohort

The population cohort consisted of 357,903 live newborns, based on the number of births in Canada during the 2022–2023 period [18]. At the time of analysis, 10 provinces in Canada were screening for SMA, covering 95.56% of the newborn population [19]. Our model includes both screened and unscreened newborns, with the vast majority (95.56%) being screened for SMA. Newborns identified through screening could be presymptomatic or symptomatic at the time of screening. Those not identified through NBS were diagnosed clinically and were symptomatic at the time of diagnosis.

#### Screening/decision tree parameters

A dried blood spot (DBS) screening sample is taken within 48 h of birth and analyzed using MassArray technology to detect homozygous deletion of the *SMN1* gene, which accounts for 95% of SMA cases. Screen positive results are confirmed using a digital droplet polymerase chain reaction (ddPCR) test, which targets specific sequence differences unique to each gene and quantifies them, allowing for the determination of *SMN2* copy number [20]. Some *SMN1* point mutations may not be identified through the initial DBS screening, estimated to occur in 5% of SMA cases [3]. We therefore assume a false negative rate of 5%, with these cases missed by NBS identified through clinical presentation. Since NBS for SMA in Canada has only recently been implemented, we used the commonly referenced incidence rate of 1 in 10,000 newborns for those diagnosed with SMA [3]. Following the implementation of SMA screening in Ontario in January 2020, no false positives have been reported by Newborn Screening Ontario (NSO). This is supported by the high specificity of the two-tiered algorithm and the requirement for molecular confirmation prior to diagnosis or treatment. The use of this sequential testing strategy substantially reduces the likelihood of false positives, a result that has been consistent with reports in similar programs internationally [3, 14].

Survival and disease severity vary widely based on SMA type and *SMN2* gene copy number [1]. Patients who underwent screening are classified by their *SMN2* gene copy number and can be either symptomatic or presymptomatic. According to NSO data on SMA screening at the time of this study beginning in 2020, the distribution of *SMN2* copy numbers among Ontario SMA patients is as follows: 48% have 2 copies, 35% have 3 copies, and 17% have 4 copies. Among these, 14% presented symptomatic at screening. The distribution of SMA types for those who present clinically was determined from clinical expert’s opinions, with percentages of 60%, 25%, and 15% for SMA types 1, 2, and 3, respectively.

Upon review of NSO data and consultation with clinical experts, we observed scenarios where parents of SMA-diagnosed patients had declined treatment. Consequently, we incorporated an arm into the decision tree where 1% of patients would decline treatment in both the screening and non-screening arms.

We also included bridge therapy treatment in the decision tree, a technique currently being implemented in treating Canadian SMA patients diagnosed through NBS [21]. There are two distinct scenarios for bridge treatment implementation. Firstly, when a patient has 2 copies of the *SMN2* gene and is symptomatic at birth, they receive one dose of nusinersen in the first month of life, followed by treatment with OA. This was estimated to occur in 99% of NBS patients presenting as symptomatic at screening, based on expert consultation. The second scenario involves SMA patients born preterm or who are antibody positive for the adeno-associated serotype 9 (AAV9) vector [21, 22]. In this case, they receive four doses of nusinersen before treatment with OA. In the NBS arm, this scenario was estimated to occur in 5% of presymptomatic patients, while the remaining 95% of presymptomatic patients in the NBS arm were treated with OA only. However, bridge therapy did not apply to the non-screening arm, as these patients present clinically later in life.

For patients with 4 copies of the *SMN2* gene, their treatment in the model follows current protocol in Canada, based on drug approvals. Patients with 4 *SMN2* copies are not approved for treatment in Canadian provinces or territories, except in the province of Quebec. Therefore, in the *SMN2* 4 copy arm, 23% of patients were eligible for treatment, representing the population of Quebec [23]. Based on expert opinion 60% of this population would receive treatment, with 80% treated with risdiplam and 20% treated with nusinersen.

**Fig. 1:**
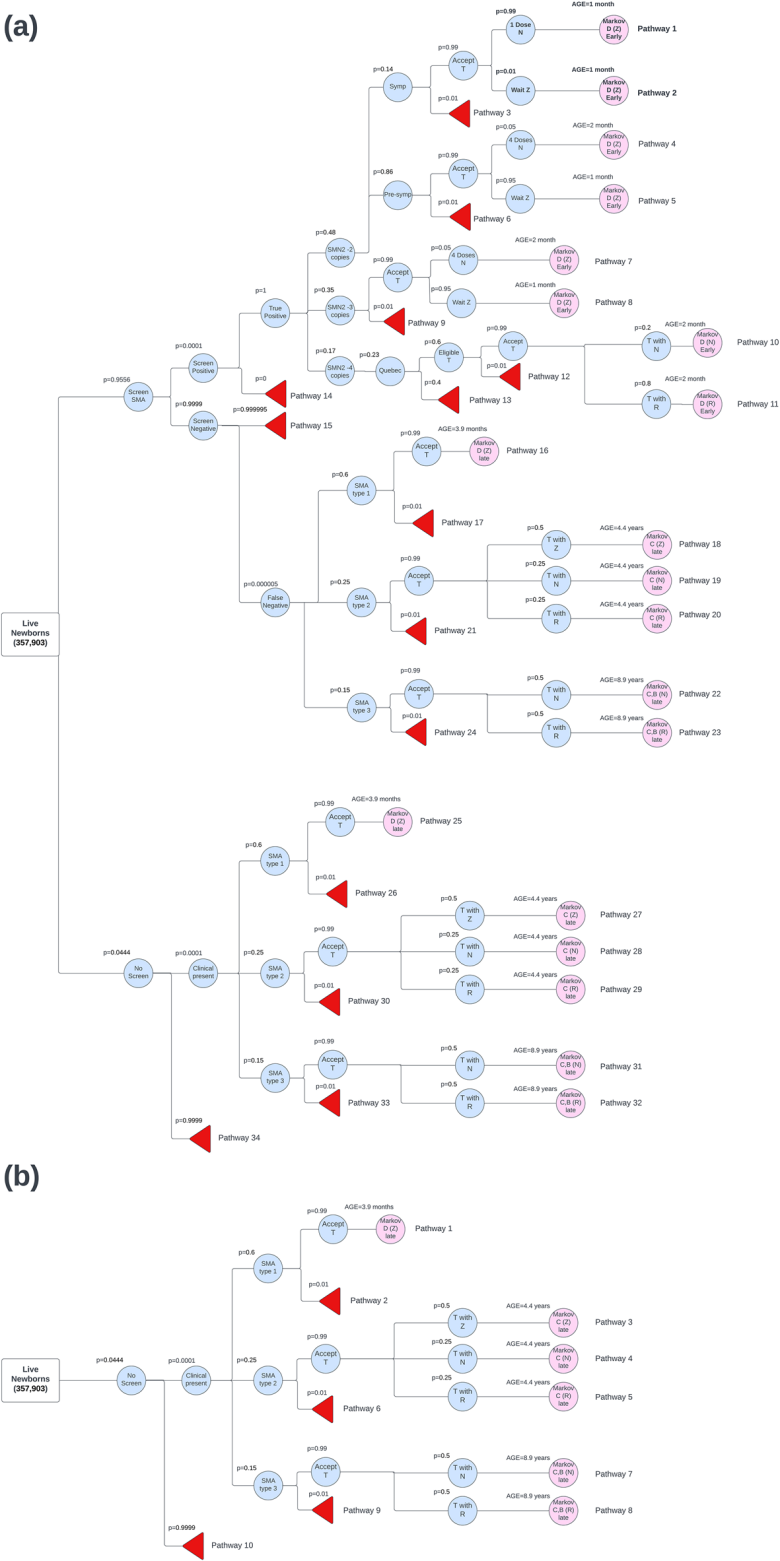
(**a**) Decision‑tree model where newborn screening–based detection exists with a portion of spinal muscular atrophy (SMA) patients clinically presenting in the Canadian birth cohort (*n* = 357 903) (**b**) Decision‑tree model where newborn screening-based detection does not exist and all SMA patients clinically present in the Canadian birth cohort (*n* = 357 903). SMA: spinal muscular atrophy; NBS: newborn screening; Symp: symptomatic; Pre-symp: Pre-symptomatic; SMN2: survival motor neuron 2; T: treatment; N: Nusinersen; Z: Zolgensma (OA); R: Risdiplam

For patients in the clinical diagnoses arm of the decision tree, the treatment protocol varies. In the model, all patients with SMA type 1 receive treatment with OA. For patients with SMA type 2, 50% were treated with OA, 25% with nusinersen, and 25% with risdiplam. For patients with SMA type 3, 50% received nusinersen, and 50% received risdiplam. These percentages were based on consultation with pediatric neurologists. The results of progress through the decision tree determine the starting health state for individuals as they enter the Markov model (Fig. 1).

### Clinical inputs

**Fig. 2:**
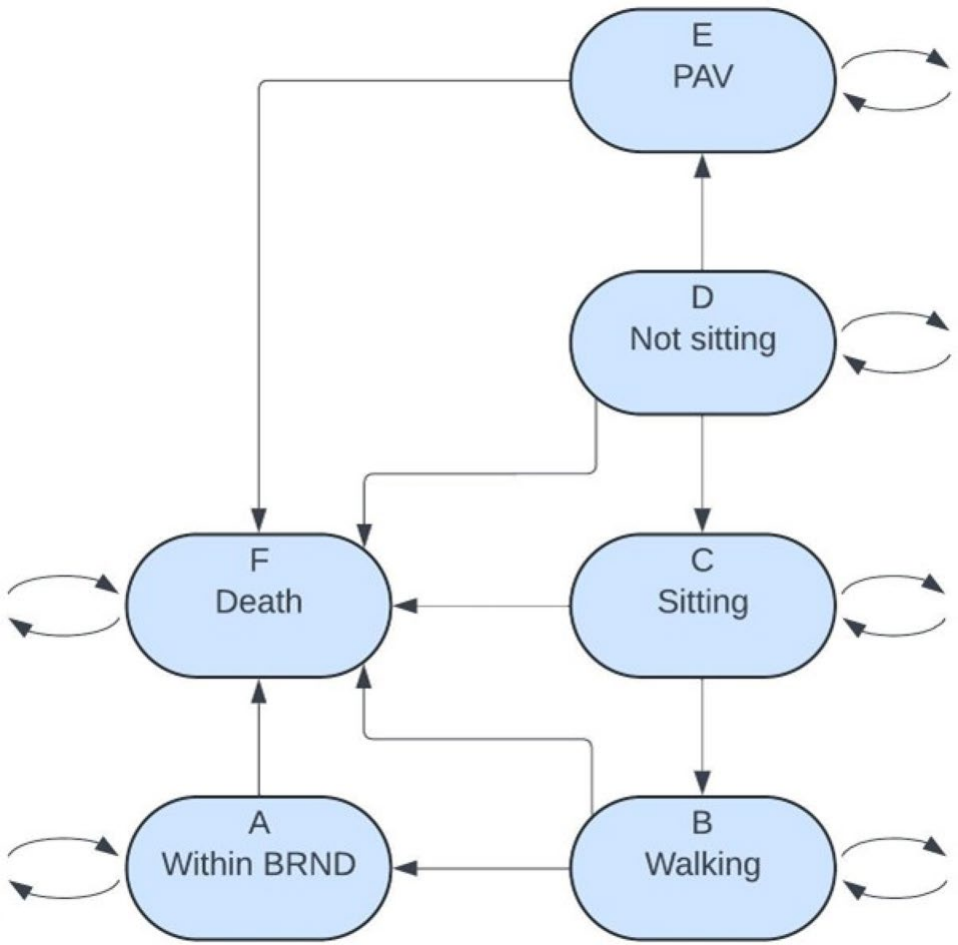
SMA Markov model diagram. SMA: spinal muscular atrophy; BRND: broad range of normal development; PAV: permanent assisted ventilation

The Markov model was developed using short-term clinical trial data and long-term survival estimates. Clinical trial data on the treatment of SMA with DMTs approved by Health Canada were utilized to estimate clinical effectiveness. Transition probabilities between the Markov states, which represent motor milestone development, were calculated using results from relevant trials [7, 8, 15, 16]. We derived hazard rates from the clinical trial and long-term survival data and these hazard rates are entries in the transition-rate matrix, $\:Q\left(t\right)$, where $\:t\:$ is the time in months. This transition-rate matrix, $\:Q\left(t\right)$, is located in Table A1. We used the formula $\:P\left(s\right)=EXP\left(Q\left(t\right)\:s\right)$ to receive the transition-probability matrix, $\:P\left(s\right)$. $\:P\left(s\right)$ is the transition-probability matrix $\:s\:$time steps forward and $\:EXP\left(Q\left(t\right)\:s\right)$ is the matrix exponential of the matrix $\:Q\left(t\right)s$. A one-month time step in the Markov model is given by $\eqalign{& X\left( {t + 1} \right) = X\left( t \right) \cdot P\left( 1 \right) = \cr& X\left( t \right) \cdot EXP\left( {Q\left( t \right) \cdot 1} \right) = X\left( t \right)EXP\left( {Q\left( t \right)} \right) \cr}$, where $\:X\left(t\right)$ is the Markov chain with the 6 states: A (Within BRND), B (Walking), C (Sitting), D (Not sitting), E (PAV), and F (Death). The Markov Model and health states are shown in Fig. 2. Clinical trial data informed the initial state patients entered the Markov model, and these trials determined motor milestones achievement over a patient’s lifetime. SMA natural history studies and Canadian life tables were used to ascertain the long-term survival outcomes for the patients in the model. Due to the trials enrolling only up to *SMN2* 3-copy patients, data on *SMN2* 3-copy patients was utilized in the absence of data for *SMN2* 4-copy patients.

Treatment following early detection among *SMN2* 2-copy and 3-copy patients, was modeled using data from the SPR1NT trial [8]. This trial investigated early treatment in children with *SMN2* 2 and 3 copies within the first month of life, who received treatment with OA. Based on consultation with clinical experts, it was assumed all *SMN2* 2-copy and 3-copy children diagnosed by NBS would be treated with OA during the first model cycle (first month of life). For SMN2 4-copy patients, data from the NURTURE trial was used [7]. The NURTURE trial evaluated early treatment in children with SMN2 2 and 3 copies, treated with nusinersen. We assumed SMN2 3-copy data for the SMN2 4-copy patients. To date, there are no risdiplam clinical trials that fully described the motor milestone achievement for individual patients; therefore, we assumed the efficacy of nusinersen to be equivalent to risdiplam.

NBS patients entered the Markov model in health state D (not-sitting), as they are not sitting at the time of diagnosis. This approach is consistent with previous studies [13, 14]. To more accurately estimate the fatality rate, lifetime costs and quality-adjusted life years (QALYs) in health state D, if the age of a patient inside of health state D was within the 99th percentile of motor milestone achievement for sitting (approx. 9 months), then the fatality rate, costs and QALYs of health state A (BRND) were applied [12]. If the age of a patient inside of health state D was outside this percentile, then the not-sitting health state D fatality rate, costs and QALYs would be applied. Similarly for health state C, if the age of a patient inside of health state C was within the 99th percentile of motor milestone achievement for walking (approx. 18 months), then the fatality rate, costs and QALYs of health state A were applied [12]. If outside this percentile, then the fatality rate, costs and QALYs of health state C would be applied. Since health state A is the walking BRND state, if a patient remains in the walking B state after 18 months of age, the walking health state B costs and QALYs of health state B are applied. Otherwise, if the age is under 18 months in the B state, then the costs and QALYs of health state A were applied. The fatality rate of health state A and B are assumed to be the same. A diagram illustrating the assumed fatality rate, costs and QALYs within health states based on the age of the patient and motor milestone expected achievement is included in the supplementary appendix, item 2.

Patients who clinically present in the model (either not screened or missed by NBS), are treated based on their SMA subtype. All patients with SMA type 1 receive treatment with OA, utilizing clinical trial data from the STR1VE trial, and enter the Markov Model in health state D (not-sitting) [16]. The STR1VE trial enrolled patients diagnosed with SMA type 1, with a mean age of 3.9 months. For patients with SMA type 2 and type 3, clinical trial data was taken from the CS2/CS12 study. The CS2/CS12 trial enrolled patients with SMA type 2 and 3 aged between 2 and 15 years, who were treated with nusinersen [13]. There was no motor milestone achievement data for individual patients from clinical trials assessing late treatment with OA or risdiplam; therefore, we assumed the treatment outcomes of nusinersen to be equal for OA and risdiplam. The average age at which treatment with nusinersen begins is 4.4 years for SMA type 2 and 8.9 years for SMA type 3 in the CS2/CS12 trial. These ages are used as the starting ages for the first cycle in the Markov model for patients with SMA type 2 and SMA type 3. All SMA type 2 patients entered in health state C (sitting), and 24% of the SMA type 3 patients entered in health state C (sitting) and 76% of the SMA type 3 patients entered in health state B (walking) respectively, based on the CS2/CS12 clinical trial motor milestone data. Following the same procedure as the NBS group, the clinical presentation group also has the lifetime costs and QALYs adjusted based on their age and the 99th percentile of motor milestone achievement information. For the clinically presenting SMA type 1 patients, the BRND (State A) utility are applied for the first 3.9 months before the SMA type 1 patient enters the Markov model in the not-sitting state. For the clinically presenting SMA type 2 patients, the BRND (State A) utility are applied for the first 18 months and then the Sitting (State C) utility are applied for the next 35 months before the SMA type 1 patient enters the Markov model in the sitting state. For the clinically presenting SMA type 3 patients, the BRND (State A) utility are applied for the first 18 months, and then for the next 89 months 24% of the utility are from the Sitting (State C) and 76% of the utility are from the Walking (State B) before 24% of the SMA type 3 patients enters the Markov model in the sitting state and 76% of the SMA type 3 patients enters the Markov model in the walking state. Longer-term survival data was determined for each health state, which included patients at risk for transition to PAV or death. For the BRND and walking health states, the transition to death was determined based on Canadian life tables from Statistics Canada [24]. Transition from the sitting health state to death was based on the natural history of SMA type 2/3 patients, as published by Zerres et al. [25]. Transition from not sitting to death is derived from two natural history studies of patients with SMA type 1, as reported by Finkel et al. and Kolb et al. [26, 27]. Lastly, the transition from “PAV” to death was sourced from Gregoretti et al. [28].

### Cost inputs

The cost of NBS for SMA is divided into treatment and health state costs. Treatment costs include the DBS screening test, any additional genetic testing for SMA-positive patients, and the costs associated with DMTs.

The cost of the DBS screening test was obtained from the Ontario Schedule of Benefits for Laboratory Services, with a listed cost of $10.76 CAD per neonate [29]. The majority of this cost is attributed to patient documentation and collection. The genetic test used to confirm an abnormal DBS test at NSO is ddPCR, which costs $250 CAD per test, based on expert opinion from the NSO Laboratory head. The costs of DMTs were sourced from Canada’s Drug Agency (CDA) reimbursement reviews: OA at $2,910,500 CAD per single dose, nusinersen at $118,000 CAD per dose, and risdiplam at $193.97 CAD per milligram [30–32].

Treatment costs for nusinersen included the 4 loading doses within the first 63 days of the initial dose, followed by one dose every 4 months for the patient’s lifetime. Additionally, we incorporated the cost of bridge therapy, which includes a single dose of nusinersen before subsequent treatment with OA, as demonstrated in screen-positive SMA patients who presented symptomatic at birth. Bridge therapy costs were also applied to a small percentage (5%) of patients who are antibody-positive or born pre-term, where they received 4 loading doses before treatment with OA [21, 22].

Treatment costs for risdiplam vary over the first two years. For those who received risdiplam under the age of two, the annual cost was $93,456 CAD. For those over the age of two, requiring a higher dosage, the annual cost was $354,000 CAD [32]. Full screening and treatment costing parameters are included in Table 1.

Health state costs were structured using the five included health states, reflecting the WHO motor milestone achievements of children, and the exhaustive state of death. These costs were determined through a literature search on resource utilization by patients with SMA and were broken down into monthly costs per health state, to reflect the model cycle length.

The most severe health state is State E (PAV) which includes costs related to patients with SMA type 1 who require mechanical ventilation for more than 16 h daily. The next health state is State D (not-sitting) where SMA type 1 costs were applied, as this state represents the expected natural history motor milestone outcome of patients with SMA type (1) The third health state is State C (sitting) where SMA type 2 costs were applied, as this state represents the expected natural history motor milestone achievement for patients with SMA type (2) The fourth health state is State B (walking) where SMA type 3 costs were applied, as this state represents the expected motor milestone achievement of SMA type 3 patients. The final health state is state A BRND where we assumed, based on expert opinion, the cost of two neurologist visits per year.

Costs included in the health states were divided into direct health system costs, direct out of pocket costs paid by patients or caregivers, and indirect costs. Health system costs consisted of practitioner visits, hospitalization in the first year and subsequent years, ambulatory care, emergency department visits, artificial nutrition, and permanent assisted ventilation [33–40]. Costs to patients and caregivers include travel to SMA appointments, allied health professional services, and assistive devices [35]. Indirect costs considered lost potential earnings due to SMA diagnosis, including caregiver productivity loss throughout the model duration and patient productivity loss beginning at 18 years of age [35, 40]. The monthly and annual costs per health state can be found in Table 1. The full breakdown of health state costs can be found in the supplementary appendix.

### Utility values

Each health state included in the model was assigned a utility value to reflect the quality of life associated with time spent in that health state. Patients accrue health benefit for time spent in a health state, where one QALY is equal to a full year lived in full health. Utility values for each health state are obtained from Canadian literature on the quality of life of patients with SMA and are reported in Table 1 [35, 41–43]. The disutility values are Gamma distributed to allow for the possibility of negative utility values, which indicates a health state condition worse than death. The PAV state, Not Sitting, and Sitting state are the health states that primarily have the possibly of negative utility values from the distributions listed in Table 1.

**Table 1:** Model inputs

Parameter	Value	Distribution (parameters)	Source
**Canadian SMA Inputs**			
Live # of newborns in Canada	357,903	-	[18]
Screened for SMA	0.9556	-	[17]
Not screened for SMA	1-0.9556	-	[17]
SMA Incidence	0.0001	$$\:\text{B}\text{e}\text{t}\text{a}(a=6.57,\:b=5.30\text{E}4)$$	[3]
Quebec Population Percentage	0.23	-	[23]
Accept treatment	0.99	-	Clinical Expert
Decline treatment	0.01	-	Clinical Expert
**NBS Inputs**			
*SMN2* copy numbers (detected with NBS)			
*SMN2–2* copies	0.48	$${\rm{Dirichlet}}({\alpha _1} = 18,\,{\alpha _2} = 13,{\alpha _3} = 6)$$	NSO Data
*SMN2–3* copies	0.35	$${\rm{Dirichlet}}({\alpha _1} = 18,\,{\alpha _2} = 13,{\alpha _3} = 6)$$	NSO Data
*SMN2–4* copies	0.17	$${\rm{Dirichlet}}({\alpha _1} = 18,\,{\alpha _2} = 13,{\alpha _3} = 6)$$	NSO Data
*SMN2–2* copies (symptomatic)	0.14	$$\:Beta(a=0.177,\:b=1.09)$$	NSO Data
False negative rate	0.000005	-	[3]
True negative	1-0.000005	-	[3]
Antibody positive/pre-term (bridge)	0.05	-	Clinical Expert
Symptomatic bridge therapy	0.99	-	Clinical Expert
Quebec *SMN2–4* copies eligible for treatment	0.6	-	Clinical Expert
Quebec *SMN2–4* copies not eligible for treatment	0.4	-	Clinical Expert
**Clinical presentation inputs**			
SMA types (detected without NBS)			
Type 1 SMA	0.6	-	Clinical Expert
Type 2 SMA	0.25	-	Clinical Expert
Type 3 SMA	0.15	-	Clinical Expert
**Costing Parameters**			
*Screening costs*			
Cost of first-tier screening test	$10.76	$$\:\text{G}\text{a}\text{m}\text{m}\text{a}(\alpha\:=3.07,\:\sigma\:=3.50)$$	[29]
Cost of second-tier genetic testing	$250	-	NSO Lab Head
*Treatment costs per dose*			
Nusinersen	$118,000	-	[31]
Onasemnogene abeparvovec	$2,910,500	-	[30]
Risdiplam (per mg)	$193.97	-	[32]
PAV (State E) monthly cost*	$15,508	$$\:\text{G}\text{a}\text{m}\text{m}\text{a}(\alpha\:=44.4,\:\sigma\:=349)$$	[33–40]
Not-sitting (State D) monthly cost	$7,758	$$\:\text{G}\text{a}\text{m}\text{m}\text{a}(\alpha\:=44.4,\:\sigma\:=174)$$	[33–39]
Sitting (State C) monthly cost	$6,906	$$\:\text{G}\text{a}\text{m}\text{m}\text{a}(\alpha\:=44.4,\:\sigma\:=155)$$	[33–39]
Walking (State B) monthly cost	$3,723	$$\:\text{G}\text{a}\text{m}\text{m}\text{a}(\alpha\:=44.4,\:\sigma\:=83.8)$$	[33–39]
BRND (State A) monthly cost**	$16.67	$$\:\text{G}\text{a}\text{m}\text{m}\text{a}(\alpha\:=44.4,\:\sigma\:=0.375)$$	[33]
**Utility Values**			
PAV (monthly)	0.00/12	$$1 - {\rm{Gamma}}(\alpha = 2.17{\rm{e}}4,\,\sigma = 4.61{\rm{e}} - 5)$$	[41]
Not-sitting (monthly)	0.32/12	$$1 - {\rm{Gamma}}(\alpha = 2.18{\rm{e}}3,\,\sigma = 4.46{\rm{e}} - 4)$$	[35]
Sitting (monthly)	0.46/12	$$1 - {\rm{Gamma}}(\alpha = 2.52{\rm{e}}3,\,\sigma = 3.82{\rm{e}} - 4)$$	[35]
Walking (monthly)	0.65/12	$$1 - {\rm{Gamma}}(\alpha = 2.92{\rm{e}}3,\,\sigma = 3.24{\rm{e}} - 4)$$	[35]
BRND under age 18 years (monthly)	0.92/12	$$1 - {\rm{Gamma}}(\alpha = 8.52{\rm{e}}3,\,\sigma = 1.08{\rm{e}} - 4)$$	[42]
BRND age 18 years or older (monthly)	0.864/12	$$1 - {\rm{Gamma}}(\alpha = 8.61{\rm{e}}3,\,\sigma = 1.08{\rm{e}} - 4)$$	[43]

SMA: spinal muscular atrophy; NBS: newborn screening; SMN2: survival motor neuron 2; NSO: Newborn Screening Ontario; PAV: permanent assisted ventilation; BRND: broad range of normal development

*Health state monthly cost represents costs after the first year of diagnosis, costs incurred in the first year are higher than the costs after the first year

**BRND health state cost based on the assumption of 2 neurologist visits per year

### Uncertainty

Uncertainty in the model inputs was addressed using probabilistic and deterministic sensitivity analyses. Results of the probabilistic sensitivity analysis (PSA), based on 1,000 model simulations, are presented as the base case analysis. Additional deterministic sensitivity analysis was conducted for key parameters by systematically varying key input parameters to identify the parameters which had the highest impact on the model [44].

### Software

The analysis was completed using RStudio version 2023.12.0. The package ‘juicr’ and the method *GUI_juicr* were used to extract the data from the relevant clinical trials [7, 8, 15, 16]. The method *flexsurvreg* in the package ‘flexsurv’ was used to fit six different survival functions (exponential, Weibull, Gompertz, log-logistic, log normal, generalized gamma) to the clinical trial data. The *AICc* method in the package ‘gamlr’ was used to determine which of the six different survival functions fitting results had the minimum corrected AIC value. The corrected AIC value was used since the number of parameters to be fit in the survival functions were less than the number of observations divided by 40 [45]. The survival function that had the minimum corrected AIC value was selected as the best fit survival function to the data. The affine invariant ensemble MCMC algorithm was used to fit a Weibull survival function to the long-term survival studies [46]. The hazard function corresponding to the fitted survival function was used as the transition rate. The method *rdirichlet* in the package ‘extraDistr’ was used to generate random samples from the Dirichlet distribution for the probability of patients being *SMN2*-2 copies, *SMN2*-3 copies, and *SMN2*-4 copies. The method *expm* in the ‘expm’ package was used to $\:Q\left(t\right)$. The method *progress* in the package ‘svMisc’ was used to display the progress level of long-running tasks in RStudio. Each of the parameters within the best fit hazard function had a mean and covariance matrix estimated. The method *mvrnorm* in the ‘MASS’ package was used to generate multivariate normal random samples for the parameters within the hazard function given the parameters’ estimated mean vector and covariance matrix.

## Results

### Screening results

We estimated that 37.1 (95% CI: 15.0 to 70.7) newborns will be identified by NBS, based on the 2022–2023 cohort of 357,903. While the other 3.3 (95% CI: 3.3 to 3.3) will present clinically each year.

### Probabilistic sensitivity analysis results

The PSA was run with 1000 iterations. The distributions varied in the model were derived from existing literature of SMA screening and treatment and these distributions can be found in Table 1 and in Table A1.

**Fig. 3:**
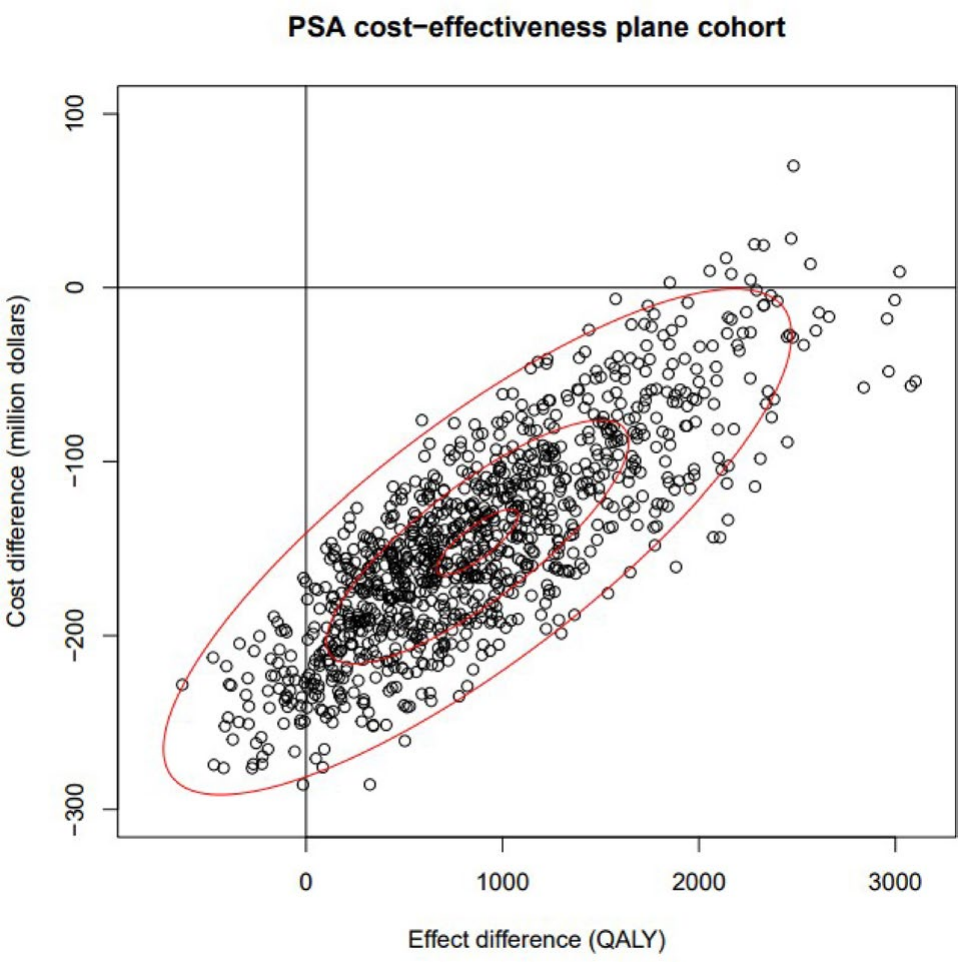
PSA cost-effectiveness plane, PSA: probabilistic sensitivity analysis; QALY: quality‑adjusted life year

The PSA results indicated that NBS and early treatment is dominant compared to no NBS and late treatment with a mean incremental cost difference of CAD -$146,187,000 (95% CI: -249,773,777 to − 17,890,034) and a mean incremental QALY benefit of 872 QALYs (95% CI: -193 to 2,328). This was reflected in the mean incremental cost-effectiveness ratio (ICER) which is -$173,572/QALY. The Incremental cost-effectiveness scatterplot (Fig. 3) shows most points fall in the southeast quadrant meaning that in most cases NBS will be less costly and more effective. The net monetary benefit (NMB) is given by the equation

$$\eqalign{& NMB\, = \,\lambda \Delta E\, - \,\Delta C\, = \cr& \lambda \left( {QALY\,NBS\, - \,QALY\,no\,NBS} \right)\, - \cr& \left( {Cost\,NBS\, - \,Cost\,no\,NBS} \right) \cr} $$

where $$\:\lambda\:\:$$ is the willingness to pay for one QALY [47]$$\:NMB>0\:$$, then the NBS is beneficial. If $$\:NMB<0\:$$, then the NBS is not beneficial. Given willingness to pay values of $1,000, $10,000, and $50,000, the NMB is found to be $147,059,934 (95% CI: $20,409,793 to $249,645,778), $154,914,379 (95% CI: 38,684,079 to 248,338,153), and $189,823,026 (95% CI: 113,519,660 to 258,439,379), respectively. NBS is beneficial for the willingness to pay values of $10,000 and $50,000, and the mean NMB for the willingness to pay value of $1,000 indicates that NBS is beneficial. Overall, the PSA results indicate that screening and early treatment for SMA is both cost-saving and more effective than not screening and late treatment. The PSA results are displayed in Table 2.

To support comparability with other studies, we also conducted a secondary analysis from the public payer perspective. As shown in Appendix Table A2, the results remained favorable, with an ICER of–$110,703 per QALY gained. The NMB at a willingness-to-pay threshold of $50,000 per QALY was $116 million, indicating that newborn screening for SMA remains cost-effective even when indirect costs are excluded. The full public payer results table is available in the Appendix Figure A2. Additionally, Appendix Figure A1 illustrates the incremental cost-effectiveness scatterplot from the public payer perspective PSA, showing that the majority of simulations fall within the southeast quadrant reinforcing that screening is both more effective and less costly from the public payer perspective.

### Deterministic sensitivity analysis

The DSA was done by finding the incremental cost difference between NBS and early treatment compared to no NBS and late treatment for the 1st, 10th, 20th, 30th, 40th, 50th, 60th, 70th, 80th, 90th, and 99th percentiles of each parameter’s distribution. The distributions were the same ones as used in the PSA analysis and these distributions can be found in Table 1 and in Table A1.

**Fig. 4:**
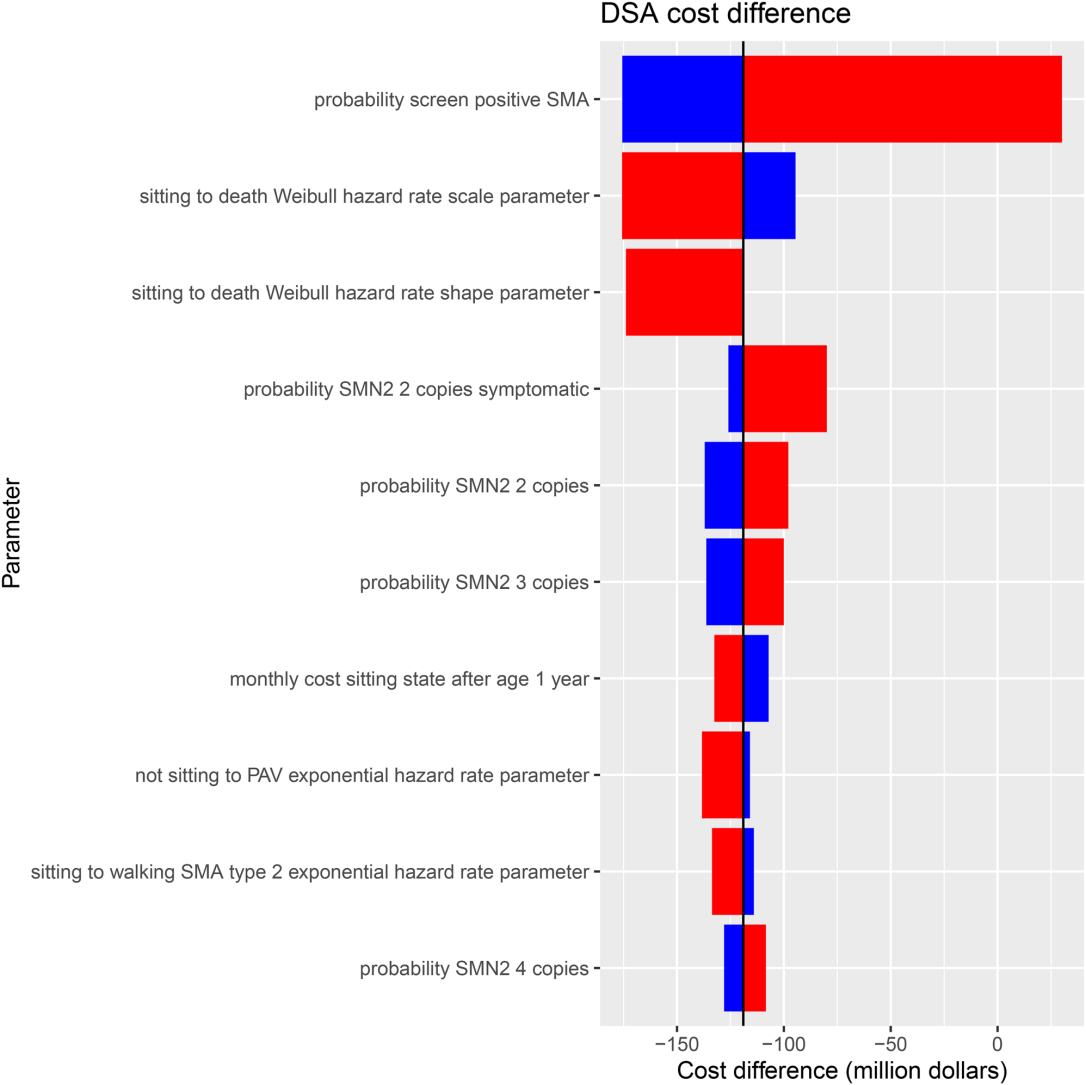
DSA tornado diagram. DSA: deterministic sensitivity analysis; SMA: spinal muscular atrophy; SMN2: survival motor neuron 2; PAV: permanent assisted ventilation

DSA results identified which model parameters had the largest impact on costs. The top ten most impactful DSA results are shown in Fig. 4. The parameters that had the largest impact on the cost difference were the probability of screening positive for SMA, the rate of moving from sitting to death, the probability of being symptomatic with 2 copies of *SMN2* and the probability of having either 2 or 3 *SMN2* copies.

**Table 2:** PSA results over a lifetime horizon and discount rate of 1.5%

	Cost (million CAD)	QALY	NMB (million CAD, willingness to pay $1,000)	NMB (million CAD, willingness to pay $10,000)	NMB (million CAD, willingness to pay $50,000)
No screening	296.9 (222.6, 360.1)	712.5025 (297.726, 1260.370)			
Screening	150.7 (77.08, 256.4)	1585.219 (640.3335, 3019.4580)	146.2 (20.41, 249.6)	154.9 (38.68, 248.3)	189.8 (113.5, 258.4)

PSA: probabilistic sensitivity analysis; CAD: Canadian dollars; QALY: quality‑adjusted life year; NMB: net monetary benefit

The scatter plot shows the cost difference against the effect difference for SMA screening and early treatment compared to no screening and late treatment. Each point represents one iteration of the PSA. Most points fall in the southeast quadrant, indicating that in most cases, SMA screening and early treatment is both less costly and more effective.

Tornado diagram illustrating the cost difference (in million CAD) from DSA. Red bars indicate high parameter value, and blue bars indicate low parameter value. The parameter “Screen Positive for SMA” has the most significant impact on overall cost, followed by “Sitting to death” and “*SMN2* copies symptomatic.

## Discussion

This model was developed using Canadian data, including the number of live births, costs, utilities, and long-term survival. To our knowledge, this is the first economic evaluation of NBS for SMA in Canada. While SMA screening has become widely adopted in Canada recently, with the final three provinces (NS, NB, PEI) adopting SMA into their NBS platforms in August 2024, questions about its cost-effectiveness remain. Additionally, this is the first SMA screening economic evaluation to incorporate bridge therapy costs into the model. In our analysis, among a newborn cohort of 357,903, NBS for SMA was associated with cost savings of $146,187,000 (95% CI: -249,773,777 to − 17,890,034) and a gain of 872 QALYs (95% CI: -193 to 2,328) compared to no NBS for SMA in the Canadian context. These findings indicate that NBS for SMA is both cost-saving and improves health outcomes, making it the dominate intervention compared to not screening. Additionally, figure A12 in the appendix indicates a high probability of cost-effectiveness across all willingness-to-pay thresholds. The earlier identification of SMA through NBS allows patients to be treated before the disease progresses, preventing irreversible loss of motor neurons. This early identification leads to patients achieving greater motor milestones, which is expected to improve health outcomes over their lifetime. The increased quality of life resulting from early identification also reduces the costs associated with living with severe SMA. The mean gain of 872 QALYs in the analysis can be attributed to the improved prognosis for patients treated early, preventing the irreversible damage seen in untreated patients. Therefore, the additional costs of investing in NBS for SMA are offset by the cost savings realized through earlier identification and immediate treatment of SMA-positive patients.

Our results demonstrate that even when parameters are varied, the model consistently shows that NBS for SMA and early treatment is cost-saving and has increased health outcomes when compared to no NBS for SMA and late treatment. The PSA scatterplot further supports this, with most points in the southeast quadrant, indicating that NBS for SMA is both less costly and more effective in most scenarios (Fig. 3). However, some scenarios in the PSA indicated that NBS might be more costly or less effective, reflecting the variability in the model. DSA results highlighted some of the parameters which had the greatest impact on costs, such as the probability of screening positive for SMA. We used the standard incidence rate of SMA commonly cited in the literature as 1 in 10,000^3^. Although the true incidence of SMA in Canada is not known, we varied this parameter using Canadian incidence rates reported in Alberta and Ontario, which were 1 in 9,401 and 1 in 27,960, respectively [5, 6].

Similar models have been completed in other countries, including England, and the Netherlands. In 2022, a cost-utility analysis from the public payer perspective in the Netherlands evaluated screening for SMA versus no screening, using a similar structure involving a decision tree and Markov model [14]. Their base case analysis found that screening for SMA resulted in a cost savings of $17,782,124 CAD and an incremental benefit of 320 QALYs, although this was among a smaller cohort of 169,680 newborns resulting in a total ICER of − 55,594 CAD/QALY. A similar model, based on the Netherlands study, was completed in 2023 using a cohort of 585,254 newborns in England from the societal perspective [13]. This study found that screening for SMA resulted in cost savings of CAD 108,213,263 and an increased QALY benefit of 529, leading to an ICER of -205,521 CAD/QALY. Our model’s results fall between those of these studies, with our mean ICER value of -$173,572/QALY. A key difference in the costing data among these studies was the high hospitalization costs reported in both the Netherlands and England studies. Our study reported moderate hospitalization costs, as early treatment should minimize hospital stays, especially after the first year of diagnosis. This assumption was also supported by our clinical experts, who agreed with the hospitalization costs used in our model. Our model was conducted from the societal perspective including both patient and caregiver lost potential earnings and other indirect costs such as travel, and out of pocket health services. The societal perspective is considered the gold standard in health economics and was best suited for this analysis [48].

There were several limitations in developing this cost-utility model. First, there was a shortage of clinical trial data for patients treated with OA who presented with clinical symptoms. Additionally, there were no risdiplam clinical trials that included individual patient data on age of achievement, making these trials ineffective for model inclusion. As a result, we used data from nusinersen trials to estimate motor milestone achievement for these clinically presenting OA patients and all risdiplam treated patients. Also, for presymptomatic *SMN2*-4 copies patients, we applied presymptomatic *SMN2*-3 copies clinical trial data to them due to a lack of clinical trial data about presymptomatic *SMN2*-4 copies patients. Furthermore, we had to extrapolate long-term effects from clinical trials that only had short-term follow-up, given DMTs have only become available recently. Data limitations also affected the quality of utility values used, as utility values for SMA patients in Canada are limited. This challenge extended to the collection of cost data, particularly regarding SMA hospitalization costs beyond the first-year post-diagnosis. Finally, we relied on expert opinion to inform the SMA treatment algorithm and costing parameters. We also assumed 100% uptake of SMA screening in provinces where it is currently offered and excluded the small proportion of families who decline screening (e.g., < 0.1% in Ontario), as decline rates were not consistently available across jurisdictions. We further assumed that individuals receiving treatment would not regress to lower-functioning health states over time, based on available clinical trial and extension data; however, long-term durability of treatment effect remains uncertain, particularly for patients treated after symptom onset.

Future research should focus on conducting clinical trials that provide individual patient motor milestone data and on extending the follow-up period to better understand the long-term outcomes of SMA patients receiving various DMTs. Additionally, more comprehensive data on the costs associated with SMA and the utility values of those diagnosed with SMA in Canada would enhance the accuracy of future economic evaluations.

The findings of this economic evaluation provide a strong basis for inclusion of screening for SMA in all Canadian NBS programs. It also provides evidence capturing the most recent techniques, such as bridge therapy, in an evolving landscape of SMA screening.

## Electronic supplementary material

Below is the link to the electronic supplementary material.

Supplementary Material 1

## Data Availability

The data supporting the findings of the study are derived from publicly available sources and clinical trial data. Screening parameters were informed by Newborn Screening Ontario data. Any additional information required to reproduce the model can be made available from the corresponding author upon reasonable request.
