# Supplementary material for: A cost-utility analysis of newborn screening for spinal muscular atrophy in Canada
Source: Orphanet J Rare Dis. 2025 Aug 13;20:428. doi: 10.1186/s13023-025-03927-6 (PMC12351935; doi:10.1186/s13023-025-03927-6)
Supplement: Supplementary file 1 — Supplementary Material 1 [file 13023_2025_3927_MOESM1_ESM.docx]

Appendix item 1: Transition rates

**Table A1** Transition rates used to obtain the transition probabilities in the Markov models

| **Transition rate** | **Symbol** | **Presymptomatic SMN2 - 2 copies (treated with Zolgensma)** | **Presymptomatic SMN2 - 3 copies (treated with Zolgensma)** | **Presymptomatic SMN2 - 4 copies (treated with Nusinersen or Risdiplam)** | **Late treatment SMA type1 (treated with Zolgensma)** | **Late treatment SMA type 2 (treated with Nusinersen, Zolgensma, or Risdiplam)** | **Late treatment SMA type 3 (treated with Nusinersen, Zolgensma, or Risdiplam)** |
| --- | --- | --- | --- | --- | --- | --- | --- |
| **Within BRND to Within BRND** | $q_{AA}$ | $q_{AA}\left( t \right)=-q_{AF}\left( t \right)$ | $q_{AA}\left( t \right)=-q_{AF}\left( t \right)$ | $q_{AA}\left( t \right)=-q_{AF}\left( t \right)$ | $q_{AA}\left( t \right)=0$ | $q_{AA}\left( t \right)=0$ | $q_{AA}\left( t \right)=-q_{AF}\left( t \right)$ |
| **Within BRND to Death** | $q_{AF}$ | If $t\leq12$,  $q_{AF}\left( t \right)=0.0001$  If $t>12$,  $q_{AF}\left( t \right)=$  Weibull hazard function, with shape 8.02 (7.70, 8.34) and scale 1.05e3 (1.05e3, 1.06e3) | If $t\leq12$,  $q_{AF}\left( t \right)=0.0001$  If $t>12$,  $q_{AF}\left( t \right)=$  Weibull hazard function, with shape 8.02 (7.70, 8.34) and scale 1.05e3 (1.05e3, 1.06e3) | If $t\leq12$,  $q_{AF}\left( t \right)=0.0001$  If $t>12$,  $q_{AF}\left( t \right)=$  Weibull hazard function, with shape 8.02 (7.70, 8.34) and scale 1.05e3 (1.05e3, 1.06e3) | $q_{AF}\left( t \right)=0$ | $q_{AF}\left( t \right)=0$ | If $t\leq12$,  $q_{AF}\left( t \right)=0.0001$  If $t>12$,  $q_{AF}\left( t \right)=$  Weibull hazard function, with shape 8.02 (7.70, 8.34) and scale 1.05e3 (1.05e3, 1.06e3) |
| **Walking to Within BRND** | $q_{BA}$ | $q_{BA}\left( t \right)=$  Log normal hazard function, with mean 3.02 (2.76, 3.28) and std dev 3.18e-1 (1.53e-1, 6.57e-1) | $q_{BA}\left( t \right)=$  Log-logistic hazard function, with shape 6.68 (4.04, 1.10e1) and scale 1.52e1 (1.33e1, 1.74e1) | $q_{BA}\left( t \right)=$  Log normal hazard function, with mean 2.57 (2.50, 2.65) and std dev 1.23e-1 (7.94e-2, 1.91e-1) | $q_{BA}\left( t \right)=0$ | $q_{BA}\left( t \right)=0$ | $q_{BA}\left( t \right)=$  Log normal hazard function, with mean 2.82 (2.58, 3.06) and std dev 4.64e-1 (2.92e-1, 7.38e-1) |
| **Walking to Walking** | $q_{BB}$ | $q_{BB}\left( t \right)=-\left( q_{BA}\left( t \right)+q_{BF}\left( t \right) \right)$ | $q_{BB}\left( t \right)=-\left( q_{BA}\left( t \right)+q_{BF}\left( t \right) \right)$ | $q_{BB}\left( t \right)=-\left( q_{BA}\left( t \right)+q_{BF}\left( t \right) \right)$ | $q_{BB}\left( t \right)=-\left( q_{BA}\left( t \right)+q_{BF}\left( t \right) \right)$ | $q_{BB}\left( t \right)=-\left( q_{BA}\left( t \right)+q_{BF}\left( t \right) \right)$ | $q_{BB}\left( t \right)=-\left( q_{BA}\left( t \right)+q_{BF}\left( t \right) \right)$ |
| **Walking to Death** | $q_{BF}$ | $q_{BF}\left( t \right)=q_{AF}\left( t \right)$ | $q_{BF}\left( t \right)=q_{AF}\left( t \right)$ | $q_{BF}\left( t \right)=q_{AF}\left( t \right)$ | $q_{BF}\left( t \right)=0$ | $q_{BF}\left( t \right)=q_{AF}\left( t \right)$ | $q_{BF}\left( t \right)=q_{AF}\left( t \right)$ |
| **Sitting to Walking** | $q_{CB}$ | $q_{CB}\left( t \right)=$  Gompertz hazard function, with shape 5.16e-1 (2.09e-1, 8.24e-1) and rate 3.20e-5 (1.97e-7, 5.20e-3) | $q_{CB}\left( t \right)=$  Log-logistic hazard function, with shape 8.34 (5.42, 1.28e1) and scale 1.49e1 (1.34e1, 1.65e1) | $q_{CB}\left( t \right)=$  Log normal hazard function, with mean 2.57 (2.50, 2.65) and std dev 1.23e-1 (7.94e-2, 1.91e-1) | $q_{CB}\left( t \right)=0$ | $q_{CB}\left( t \right)=$  Exponential hazard function, with rate 9.18e-4 (1.29e-4, 6.52e-3) | $q_{CB}\left( t \right)=$  Log normal hazard function, with mean 2.71 (2.56, 2.87) and std dev 3.19e-1 (2.28e-1, 4.47e-1) |
| **Sitting to Sitting** | $q_{CC}$ | $q_{CC}\left( t \right)=-\left( q_{CB}\left( t \right)+q_{CF}\left( t \right) \right)$ | $q_{CC}\left( t \right)=-\left( q_{CB}\left( t \right)+q_{CF}\left( t \right) \right)$ | $q_{CC}\left( t \right)=-\left( q_{CB}\left( t \right)+q_{CF}\left( t \right) \right)$ | $q_{CC}\left( t \right)=-\left( q_{CB}\left( t \right)+q_{CF}\left( t \right) \right)$ | $q_{CC}\left( t \right)=-\left( q_{CB}\left( t \right)+q_{CF}\left( t \right) \right)$ | $q_{CC}\left( t \right)=-\left( q_{CB}\left( t \right)+q_{CF}\left( t \right) \right)$ |
| **Sitting to Death** | $q_{CF}$ | $q_{CF}\left( t \right)=$  Weibull hazard function, with shape 1.91 (3.15e-1, 1.57e1) and scale 4.82e2 (3.04e2, 5.54e3) | $q_{CF}\left( t \right)=$  Weibull hazard function, with shape 1.91 (3.15e-1, 1.57e1) and scale 4.82e2 (3.04e2, 5.54e3) | $q_{CF}\left( t \right)=$  Weibull hazard function, with shape 1.91 (3.15e-1, 1.57e1) and scale 4.82e2 (3.04e2, 5.54e3) | $q_{CF}\left( t \right)=$  Weibull hazard function, with shape 1.91 (3.15e-1, 1.57e1) and scale 4.82e2 (3.04e2, 5.54e3) | $q_{CF}\left( t \right)=$  Weibull hazard function, with shape 1.91 (3.15e-1, 1.57e1) and scale 4.82e2 (3.04e2, 5.54e3) | $q_{CF}\left( t \right)=$  Weibull hazard function, with shape 1.91 (3.15e-1, 1.57e1) and scale 4.82e2 (3.04e2, 5.54e3) |
| **Not Sitting to Sitting** | $q_{DC}$ | $q_{DC}\left( t \right)=$  Log normal hazard function, with mean 2.10 (1.99, 2.21) and std dev 2.05e-1 (1.42e-1, 2.97e-1) | $q_{DC}\left( t \right)=$  Log normal hazard function, with mean 2.04 (1.94, 2.14) and std dev 1.86e-1 (1.29e-1, 2.70e-1) | $q_{DC}\left( t \right)=$  Log normal hazard function, with mean 1.89 (1.78, 2.00) and std dev 1.77e-1 (1.14e-1, 2.74e-1) | $q_{DC}\left( t \right)=$  Log normal hazard function, with mean 2.71 (2.56, 2.86) and std dev 3.30e-1 (2.23e-1, 4.89e-1) | $q_{DC}\left( t \right)=0$ | $q_{DC}\left( t \right)=0$ |
| **Not Sitting to Not Sitting** | $q_{DD}$ | $q_{DD}\left( t \right)=-\left( q_{DC}\left( t \right)+q_{DE}\left( t \right)+q_{DF}\left( t \right) \right)$ | $q_{DD}\left( t \right)=-\left( q_{DC}\left( t \right)+q_{DE}\left( t \right)+q_{DF}\left( t \right) \right)$ | $q_{DD}\left( t \right)=-\left( q_{DC}\left( t \right)+q_{DE}\left( t \right)+q_{DF}\left( t \right) \right)$ | $q_{DD}\left( t \right)=-\left( q_{DC}\left( t \right)+q_{DE}\left( t \right)+q_{DF}\left( t \right) \right)$ | $q_{DD}\left( t \right)=0$ | $q_{DD}\left( t \right)=0$ |
| **Not Sitting to PAV** | $q_{DE}$ | $q_{DE}\left( t \right)=0$ | $q_{DE}\left( t \right)=0$ | $q_{DE}\left( t \right)=0$ | $q_{DE}\left( t \right)=$  Exponential hazard function, with rate 3.24e-3 (4.56e-4, 2.30e-2) | $q_{DE}\left( t \right)=0$ | $q_{DE}\left( t \right)=0$ |
| **Not Sitting to Death** | $q_{DF}$ | $q_{DF}\left( t \right)=$  Weibull hazard function, with shape 1.32 (1.11e-1, 1.93) and scale 1.82e1 (1.19e1, 1.47e3) | $q_{DF}\left( t \right)=$  Weibull hazard function, with shape 1.32 (1.11e-1, 1.93) and scale 1.82e1 (1.19e1, 1.47e3) | $q_{DF}\left( t \right)=$  Weibull hazard function, with shape 1.32 (1.11e-1, 1.93) and scale 1.82e1 (1.19e1, 1.47e3) | $q_{DF}\left( t \right)=$  Weibull hazard function, with shape 1.32 (1.11e-1, 1.93) and scale 1.82e1 (1.19e1, 1.47e3) | $q_{DF}\left( t \right)=0$ | $q_{DF}\left( t \right)=0$ |
| **PAV to PAV** | $q_{EE}$ | $q_{EE}\left( t \right)=0$ | $q_{EE}\left( t \right)=0$ | $q_{EE}\left( t \right)=0$ | $q_{EE}\left( t \right)=-q_{EF}\left( t \right)$ | $q_{EE}\left( t \right)=0$ | $q_{EE}\left( t \right)=0$ |
| **PAV to Death** | $q_{EF}$ | $q_{EF}\left( t \right)=0$ | $q_{EF}\left( t \right)=0$ | $q_{EF}\left( t \right)=0$ | $q_{EF}\left( t \right)=$  Weibull hazard function, with shape 6.20e-1 (3.92e-1, 7.40e1) and scale 2.22e3 (7.52e2, 8.99e3) | $q_{EF}\left( t \right)=0$ | $q_{EF}\left( t \right)=0$ |

Time, *t,* is in months. The point estimate is the maximum likelihood value or maximum posterior value. The interval is the 95% confidence interval or 95% credible interval.

Appendix item 2: Public Payer Perspective PSA results

Table A2: Public Payer Perspective

|  | **Cost (million CAD)** | **QALY** | **NMB (million CAD, willingness to pay $1,000)** | **NMB (million CAD, willingness to pay $10,000)** | **NMB (million CAD, willingness to pay $50,000)** |
| --- | --- | --- | --- | --- | --- |
| *No screening* | 214.5 (183.1, 240.7) | 712.5025 (297.726, 1260.370) |  |  |  |
| *Screening* | 142.2 (71.57, 247.0) | 1585.219 (640.3335, 3019.4580) | 73.21 (-34.12, 152.6) | 81.06 (-14.55, 152.2) | 116.0 (65.49, 158.0) |

Public Payer ICER –110,702.9

Figure A1: PSA Cost-effectiveness plane (payer perspective)


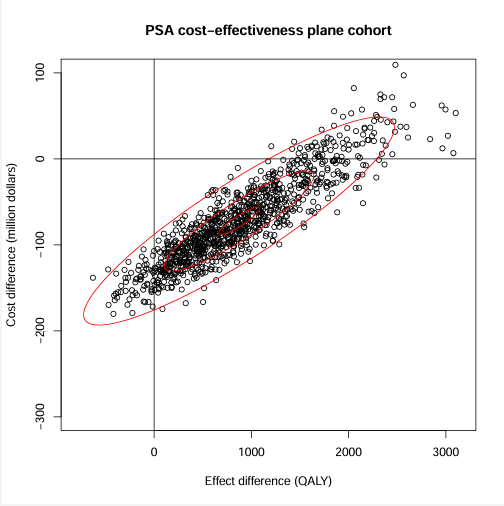


PSA = Probabilistic Sensitivity Analysis; QALY = Quality-Adjusted Life Year;

Appendix item 3: Fatality rate, cost, and utility used within health state B, C, and D based on patient age

**Figure A2**


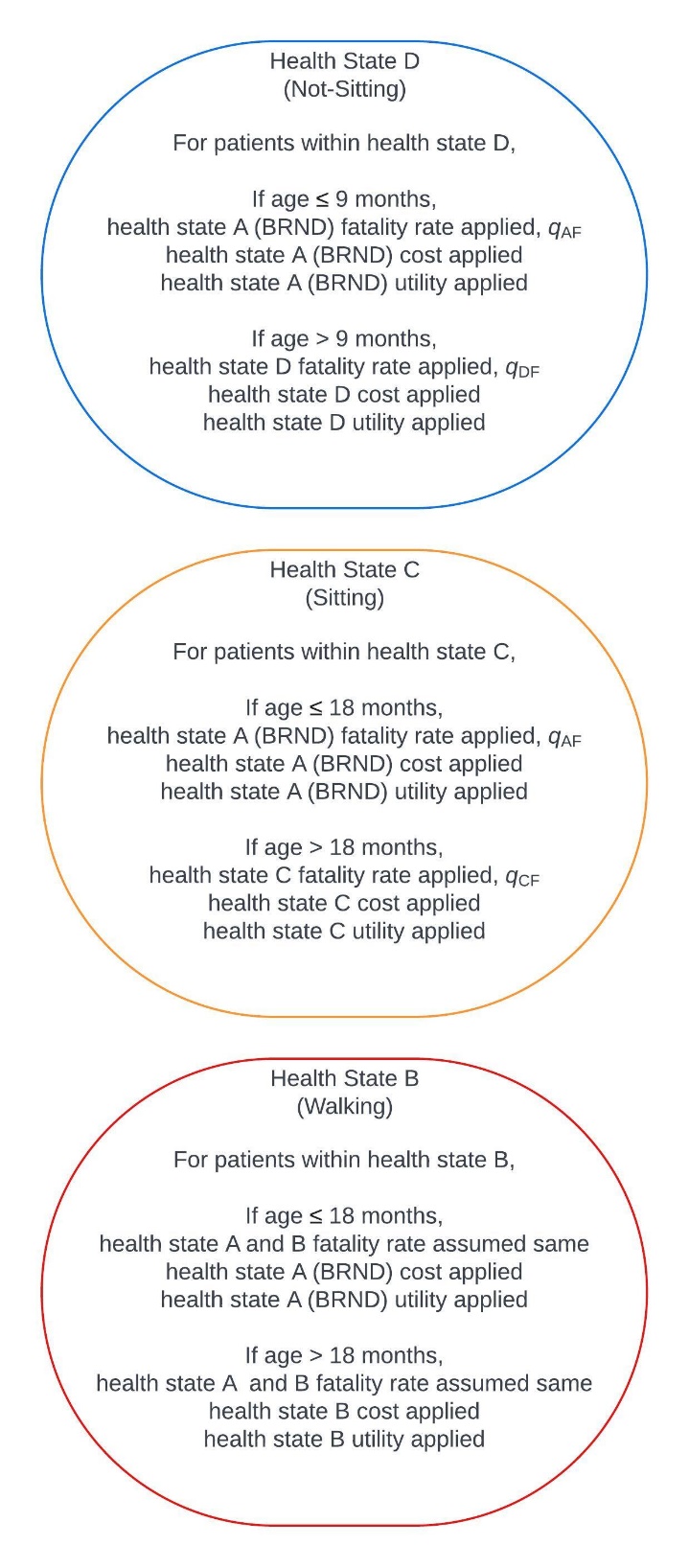


*BRND (Broad Range of Normal Development)

Appendix Item 4: Health States costing breakdown

**Table A3** Health State E (PAV) SMA Type 1 costs + PAV costs

| **Costing parameter** | **Cost** | **Time Frame** | **Cost per month** | **Source** |
| --- | --- | --- | --- | --- |
| **Direct Health System Costs** | | | | |
| Practitioner visits | $50.80 | Per month (first 4 years) | $50.80 | 1 |
| Hospitalization 1^st^ year | $29,494 | First-year after diagnosis | $2,457.83 | 2 |
| Hospitalization subsequent years | $6,866.68 | Annual cost of overnight inpatient visit | $572.23 | 7,8 |
| Ambulatory care | $5,534 | First-year after diagnosis | $461.16 | 2 |
| Emergency department visits | $383 | Annual | $31.91 | 3,4 |
| Artificial nutrition | $303 | Per month | $303 | 9 |
| Cost of invasive PAV | $7,550 | Monthly | $7,550 | 5 |
| **Out-of-pocket costs** | | | | |
| Travel | $4,270 | Annual | $355.83 | 3 |
| Allied health professional services | $14,849 | Annual | $1,237.41 | 3 |
| Assistive devices | $9,792 | Annual | $816 | 3 |
| **Indirect Costs** | | | | |
| Patient lost potential earnings | $2,513 | Per month at 18 years of age | $2,513 | 3 |
| Caregiver lost potential earnings | $4391 | Per month | $4391 | 9 |
| **Total cost per month first year post diagnosis** | $18,227 | | | |
| **PAV First Year total** | $218,724 | | | |
| **Total cost per month subsequent years** | $15,508 | | | |
| **PAV annual cost** | $186,096 | | | |

Note: Patient lost potential earnings are not included in total cost calculations prior to age 18. In the model, these indirect costs are applied only once patients reach 18 years of age, reflecting entry into the working-age population.

**Table A4** Health State D (not sitting) SMA type 1 costs

| **Costing parameter** | **Cost** | **Time Frame** | **Cost per month** | **Source** |
| --- | --- | --- | --- | --- |
| **Direct Health System Costs** | | | | |
| Practitioner visits | $50.80 | Per month (first 4 years) | $50.80 | 1 |
| Hospitalization 1^st^ year | $29,494 | First-year after diagnosis | $2,457.83 | 2 |
| Hospitalization subsequent years | $6,866.68 | Annual cost of overnight inpatient visit | $572.23 | 7,8 |
| Ambulatory care | $5,534 | First-year after diagnosis | $461.16 | 2 |
| Emergency department visits | $383 | Annual | $31.91 | 3,4 |
| Artificial nutrition | $303 | Per month | $303 | 9 |
| **Out-of-pocket costs** | | | | |
| Travel | $4,270 | Annual | $355.83 | 3 |
| Allied health professional services | $14,849 | Annual | $1,237.41 | 3 |
| Assistive devices | $9,792 | Annual | $816 | 3 |
| **Indirect Costs** | | | | |
| Patient lost potential earnings | $2,513 | Per month at age 18 years | $2,513 | 3 |
| Caregiver lost potential earnings | $4391 | Per month | $4391 | 9 |
| Total cost per month first year post diagnosis | $10,677 | | | |
| Total cost first year | $128,124 | | | |
| Total cost per month subsequent years | $7,758 | | | |
| SMA type 1 annual cost | $93,096 | | | |

Note: Patient lost potential earnings are not included in total cost calculations prior to age 18. In the model, these indirect costs are applied only once patients reach 18 years of age, reflecting entry into the working-age population.

**Table A5** Health State C (Sitting) SMA type 2 costs

| **Costing parameter** | **Cost** | **Time Frame** | **Cost per month** | **Source** |
| --- | --- | --- | --- | --- |
| **Direct Health System Costs** | | | | |
| Practitioner visits | $50.80 | Per month (first 4 years) | $50.80 | 1 |
| Hospitalization 1^st^ year | $18,086 | First-year after diagnosis | $1,507.16 | 2 |
| Hospitalization subsequent years | $5,526.84 | Annual cost of overnight inpatient visit | $460.57 | 7,8 |
| Ambulatory care | $16,102 | First-year after diagnosis | $1,341.83 | 2 |
| Emergency department visits | $214 | Annual | $17.83 | 3,4 |
| Artificial nutrition | $38 | Per month | $38 | 9 |
| **Out-of-pocket costs** | | | | |
| Travel | $5,188 | Annual | $432.33 | 3 |
| Allied health professional services | $16,102 | Annual | $1,341.83 | 3 |
| Assistive devices | $16,038 | Annual | $1,336.50 | 3 |
| **Indirect Costs** | | | | |
| Patient lost potential earnings | $937 | Per month at age 18 | $937 | 3 |
| Caregiver lost potential earnings | $3,230 | Per month | $3,230 | 9 |
| Total cost per month first year post diagnosis | $9,754 | | | |
| Total cost per year | $117,048 | | | |
| Total cost per month subsequent years | $6,906 | | | |
| SMA type 2 annual cost | $82,877 | | | |

Note: Patient lost potential earnings are not included in total cost calculations prior to age 18. In the model, these indirect costs are applied only once patients reach 18 years of age, reflecting entry into the working-age population.

**Table A6** Health State B (walking) SMA type 3 costs

| **Costing parameter** | **Cost** | **Time Frame** | **Cost per month** | **Source** |
| --- | --- | --- | --- | --- |
| **Direct Health System Costs** | | | | |
| Practitioner visits | $36.12 | Per month (first 4 years) | $36.12 | 1 |
| Hospitalization 1^st^ year | $8,582 | First-year after diagnosis | $715.16 | 2 |
| Hospitalization subsequent years | $1,842.28 | Annual cost of overnight inpatient visit | $153.52 | 7,8 |
| Ambulatory care | $8,325 | First-year after diagnosis | $693.75 | 2 |
| Emergency department visits | $151 | Annual | $12.58 | 3,4 |
| Artificial nutrition | $14 | Per month | $14 | 9 |
| **Out-of-pocket costs** | | | | |
| Travel | $4,364 | Annual | $363.66 | 3 |
| Allied health professional services | $16,143 | Annual | $1,345.25 | 3 |
| Assistive devices | $18,715 | Annual | $1,559.58 | 3 |
| **Indirect Costs** | | | | |
| Patient lost potential earnings | $684 | Per month at age 18 | $684 | 3 |
| Caregiver lost potential earnings | $1586 | Per month | $1,586 | 9 |
| Total cost per month first year post diagnosis | $5,131 | | | |
| Total cost first year | $61,572 | | | |
| Total cost per month subsequent years | $3,723 | | | |
| SMA type 3 annual cost | $44,684 | | | |

Note: Patient lost potential earnings are not included in total cost calculations prior to age 18. In the model, these indirect costs are applied only once patients reach 18 years of age, reflecting entry into the working-age population.

**Table A7** Health State A (Broad Range of Normal Development)

| **Costing parameter** | **Cost** | **Time Frame** | **Cost per month** | **Source** |
| --- | --- | --- | --- | --- |
| **Direct Health System Costs** | | | | |
| *Total health expenditure | $200 | Per year | $16.67 | 6 |
| Total cost per month | $16.67 | | | |

*Based on 2 neurologist visits per year

Appendix Item 5: Deterministic sensitivity analysis plots for each of the top ten parameters that had the most significant impact on overall cost

**Figure A3**

**
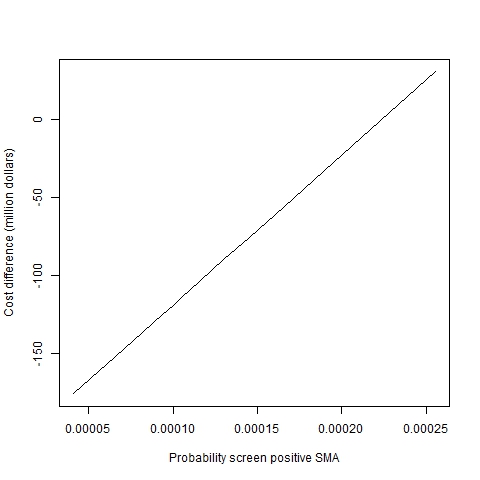
**

**Figure A4**

**
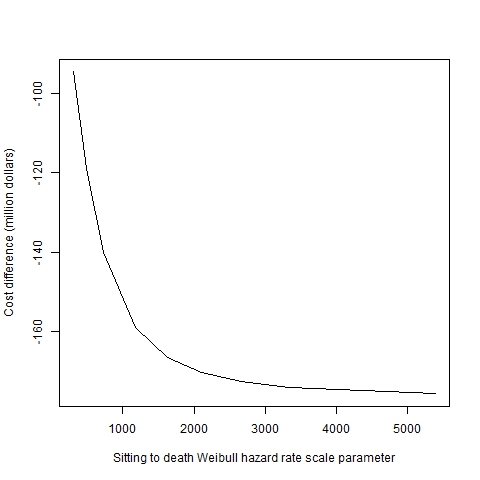
**

**Figure A5**

**
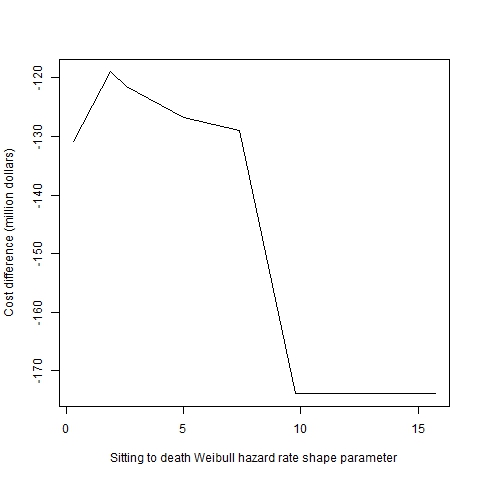
**

**Figure A6**


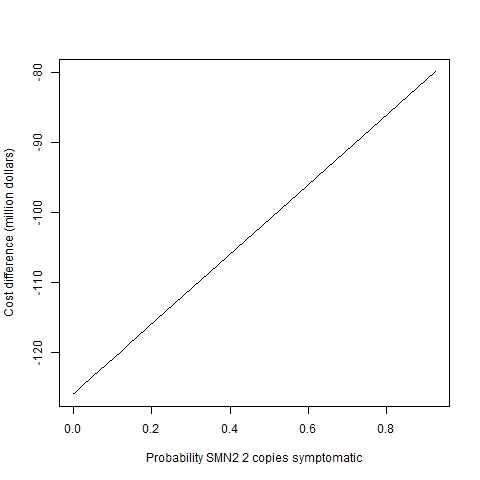


**Figure A7**

**
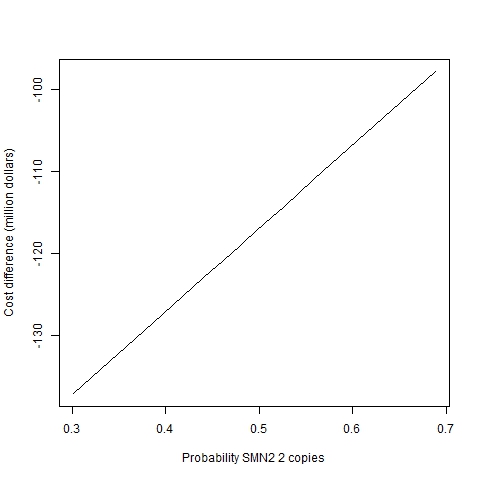
**

**Figure A8**

**
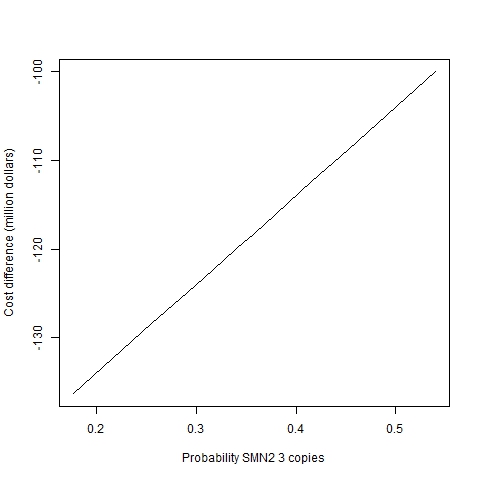
**

**Figure A9**

**
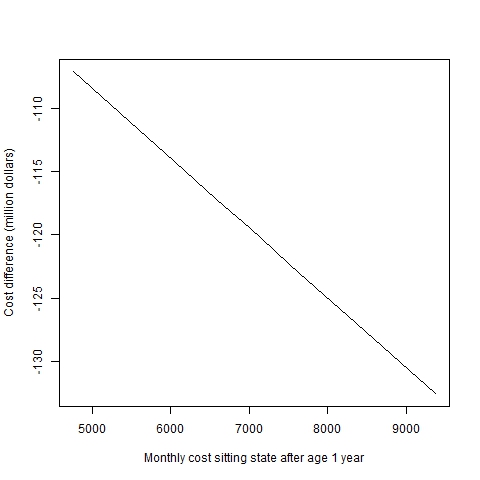
**

**Figure A10**

**
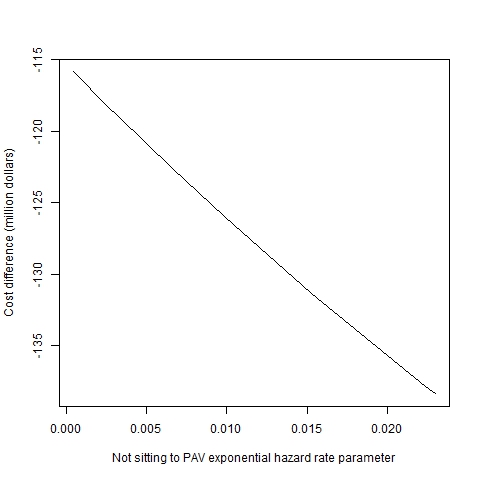
**

**Figure A11**

**
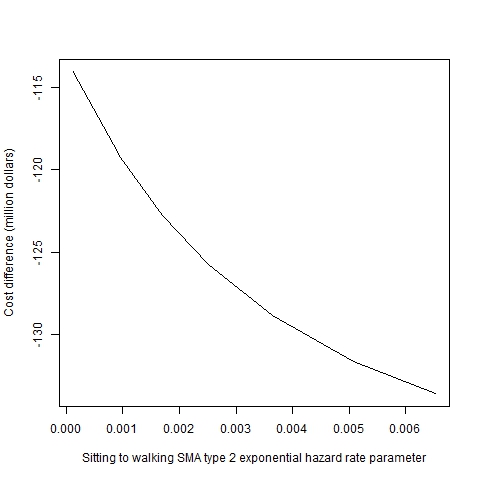
**

**Figure A12**

**
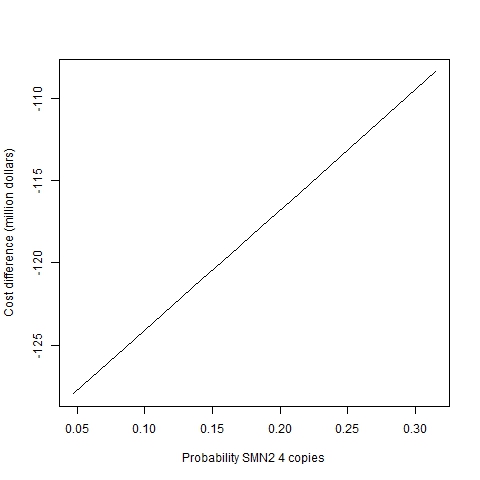
**

Appendix Item 6: Cost-effectiveness acceptability curve

Figure A13
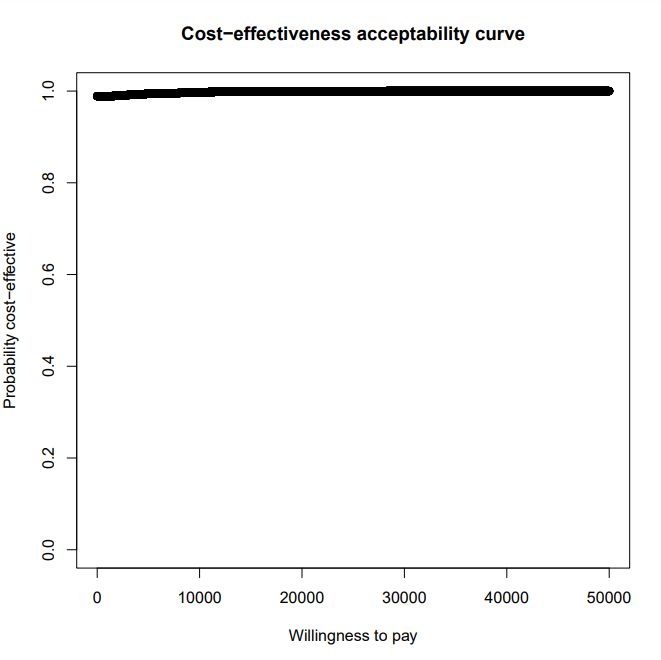

The curve illustrates the probability that SMA screening and early treatment is cost-effective compared to no screening and late treatment, across a range of willingness-to-pay thresholds. The curve indicates a high probability of cost-effectiveness across all willingness-to-pay thresholds.

References

1. McMillan HJ, Kernohan KD, Yeh E, Amburgey K, Boyd J, Campbell C, et al. Newborn Screening for Spinal Muscular Atrophy: Ontario Testing and Follow-up Recommendations. Canadian Journal of Neurological Sciences. 2021 Jul;48(4):504–11.
2. Chen G, Sharif B, Gerber B, Farris MS, Cowling T, Cabalteja C, et al. Epidemiology, healthcare resource utilization and healthcare costs for spinal muscular atrophy in Alberta, Canada. J Med Econ. 2021 Nov;24(sup1):51–9.
3. McMillan HJ, Gerber B, Cowling T, Khuu W, Mayer M, Wu JW, et al. Burden of Spinal Muscular Atrophy (SMA) on Patients and Caregivers in Canada. Journal of Neuromuscular Diseases. 2021 Jan 1;8(4):553–68.
4. Hospital spending: Focus on the emergency department.
5. Nonoyama ML, McKim DA, Road J, Guerriere D, Coyte PC, Wasilewski M, et al. Healthcare utilisation and costs of home mechanical ventilation. Thorax. 2018 Jan 26;thoraxjnl-2017-211138.
6. Weidlich D, Servais L, Kausar I, Howells R, Bischof M. Cost-Effectiveness of Newborn Screening for Spinal Muscular Atrophy in England. Neurol Ther [Internet]. 2023 May 24 [cited 2023 Jun 16]; Available from: <https://link.springer.com/10.1007/s40120-023-00489-2>
7. Sejersen T, Graham S, Ekström AB, Kroksmark AK, Kwiatkowska M, Ganz ML, et al. Healthcare resource utilisation and direct medical cost for individuals with 5q spinal muscular atrophy in Sweden. Eur J Health Econ [Internet]. 2024 Apr 20 [cited 2024 Apr 30]; Available from: <https://doi.org/10.1007/s10198-024-01678-y>
8. Cost of a Standard Hospital Stay · CIHI [Internet]. [cited 2024 Apr 30]. Available from: <https://yourhealthsystem.cihi.ca/hsp/inbrief#!/indicators/015/cost-of-a-standard-hospital-stay/;mapC1;mapLevel2;/>
9. Klug C, Schreiber-Katz O, Thiele S, Schorling E, Zowe J, Reilich P, et al. Disease burden of spinal muscular atrophy in Germany. Orphanet J Rare Dis. 2016 May 4;11:58.
